# A network diffusion approach to inferring sample-specific function reveals functional changes associated with breast cancer

**DOI:** 10.1371/journal.pcbi.1005793

**Published:** 2017-11-30

**Authors:** Sushant Patkar, Assaf Magen, Roded Sharan, Sridhar Hannenhalli

**Affiliations:** 1 Center for Bioinformatics and Computational Biology, University of Maryland, College Park, Maryland, United States of America; 2 Blavatnik School of Computer Science, Tel Aviv University, Tel Aviv, Israel; Tufts University, UNITED STATES

## Abstract

*Guilt-by-association* codifies the empirical observation that a gene’s function is informed by its neighborhood in a biological network. This would imply that when a gene’s network context is altered, for instance in disease condition, so could be the gene’s function. Although context-specific changes in biological networks have been explored, the potential changes they may induce on the functional roles of genes are yet to be characterized. Here we analyze, for the first time, the network-induced potential functional changes in breast cancer. Using transcriptomic samples for 1047 breast tumors and 110 healthy breast tissues from TCGA, we derive sample-specific protein interaction networks and assign sample-specific functions to genes via a diffusion strategy. Testing for significant changes in the inferred functions between normal and cancer samples, we find several functions to have significantly gained or lost genes in cancer, not due to differential expression of genes known to perform the function, but rather due to changes in the network topology. Our predicted functional changes are supported by mutational and copy number profiles in breast cancers. Our diffusion-based functional assignment provides a novel characterization of a tumor that is complementary to the standard approach based on functional annotation alone. Importantly, this characterization is effective in predicting patient survival, as well as in predicting several known histopathological subtypes of breast cancer.

## Author summary

Proteins that cooperate to perform a specific cellular function often interact with each other and are clustered together in a protein-protein interaction network (PIN). Thus, a protein’s function can be inferred from the prevailing functions of its network neighbors. However, the network neighborhood is highly dynamic and so, we argue, a protein’s function should be. Based on this premise, we evaluate how a protein’s function might change in a specific dynamic context of breast cancer. This novel approach uniquely reveals several functions that are significantly lost or gained in cancer. Moreover, the PIN-induced sample-specific functional activity provides a novel characterization of cancer sample that can help predict patient survival as well as several known subtypes of breast cancer.

## Introduction

Cellular functions are carried out by networks of interacting proteins [1]. Empirical data suggest that proteins that participate in the same biological process tend to interact with one another, and more broadly, tend to inhabit the same neighborhood in the protein interaction network (PIN). This *guilt-by-association* principle has been successfully applied to predict protein function, outperforming alternative methods that do not take the PIN into account [2–7]. In the following, the term ‘function’ is used broadly to represent a biological process or a pathway.

Given that a gene’s function is informed by its PIN neighborhood, and given that the network is dynamic and context-specific, it is plausible that a gene’s function can vary across different contexts, such as developmental stages, tissues, diseases, as well as during evolution. Indeed, many genes are known to function in a context-specific manner, despite being expressed broadly [8,9]. Transcription factor proteins serve as prime examples of such context-specific function [10]. For example, during *Drosophila* development, a key regulatory transcription factor *fushi tarazu* (*FTZ*) changes function from an ancestral homeotic gene (those that regulate development of specific body parts) to a pair-rule segmentation gene (regulating initial formation of the segments in a developing embryo). Notably, this functional switch involves changes in *FTZ*’s interaction partners; while in the ancestral species FTZ interacted with homeotic proteins, in drosophila it interacts with protein involved in segmentation, and thus it got co-opted into segmentation function [11]. In this work, we specifically focus on the dynamic cellular context associated with breast cancer, and potential functional changes induced by the PIN changes in breast cancer relative to the normal breast tissue.

Transformation of a normal somatic cell into a cancerous one involves a multitude of molecular changes, ultimately reflected in a major shift in the transcriptome. This general observation has motivated numerous previous studies that have proposed transcriptome-based signatures of cancer and cancer subtypes, as well as predictive models of patient survival based on the patient’s tumor transcriptome [12,13]. However, a network view of cellular functions, as discussed above, exposes limitations of a gene-wise differential expression analysis in gaining mechanistic insights into development and maintenance of tumors [14]. Accordingly, several recent studies have investigated the network-level shifts in cancer [14–16]. For instance, subnetwork changes were shown to better characterize breast cancer metastasis in comparison to changes in individual genes’ expression levels [14].

Here, we take a novel complementary approach to network-based characterization of molecular changes associated with cancer. Exploiting the principle of guilt-by-association, combined with the dynamic network topology associated with cancer, we assess whether a gene exhibits a significant qualitative change in its PIN neighborhood and, therefore, a potential change (gain or loss) in its function in cancer relative to the normal tissue. Using transcriptomic samples for 1047 breast tumors and 110 healthy breast tissues from the TCGA [17], and a comprehensive PIN taken from HIPPIE [18], we first derive sample-specific PIN and assign sample-specific functions to each gene via diffusion of functions over the network [19]. We then test for significant changes in the inferred functions between normal and cancer samples. Specifically, we estimate the sample-specific activity level of the function as the number of genes predicted to perform that function in the sample. Then, we identify functions that exhibit a substantial loss or gain in their activity levels in cancer relative to normal breast tissues.

We find several biological functions to have significantly gained or lost genes in cancer, attributed to systematic cancer-associated changes in the PIN topology; we will refer to such functions as *cancer-associated*. Notably, many of cancer-associated functions are uniquely revealed by our diffusion-based approach to functional assignment, and do not exhibit differential activity in cancer if we rely only on the *a priori* annotated genes in each sample-specific PIN. We find that the genes contributing to significant loss or gain of cancer-associated functions tend to exhibit elevated frequency of mutation as well as copy number decrease in breast cancer, suggesting that alterations in PIN may represent an alternative mechanism for functional change. We further show that the detected functional change in cancer is also reflected in a consistent association with patient survival risk. Finally, we show that using a diffusion-based functional profile of a tumor provides a better prediction of patient survival and clinical subtypes than the standard alternative, where sample-specific functional activity is quantified based only on *a priori* annotated genes in each sample-specific PIN.

## Results

### Overview of the approach

**Fig 1:**
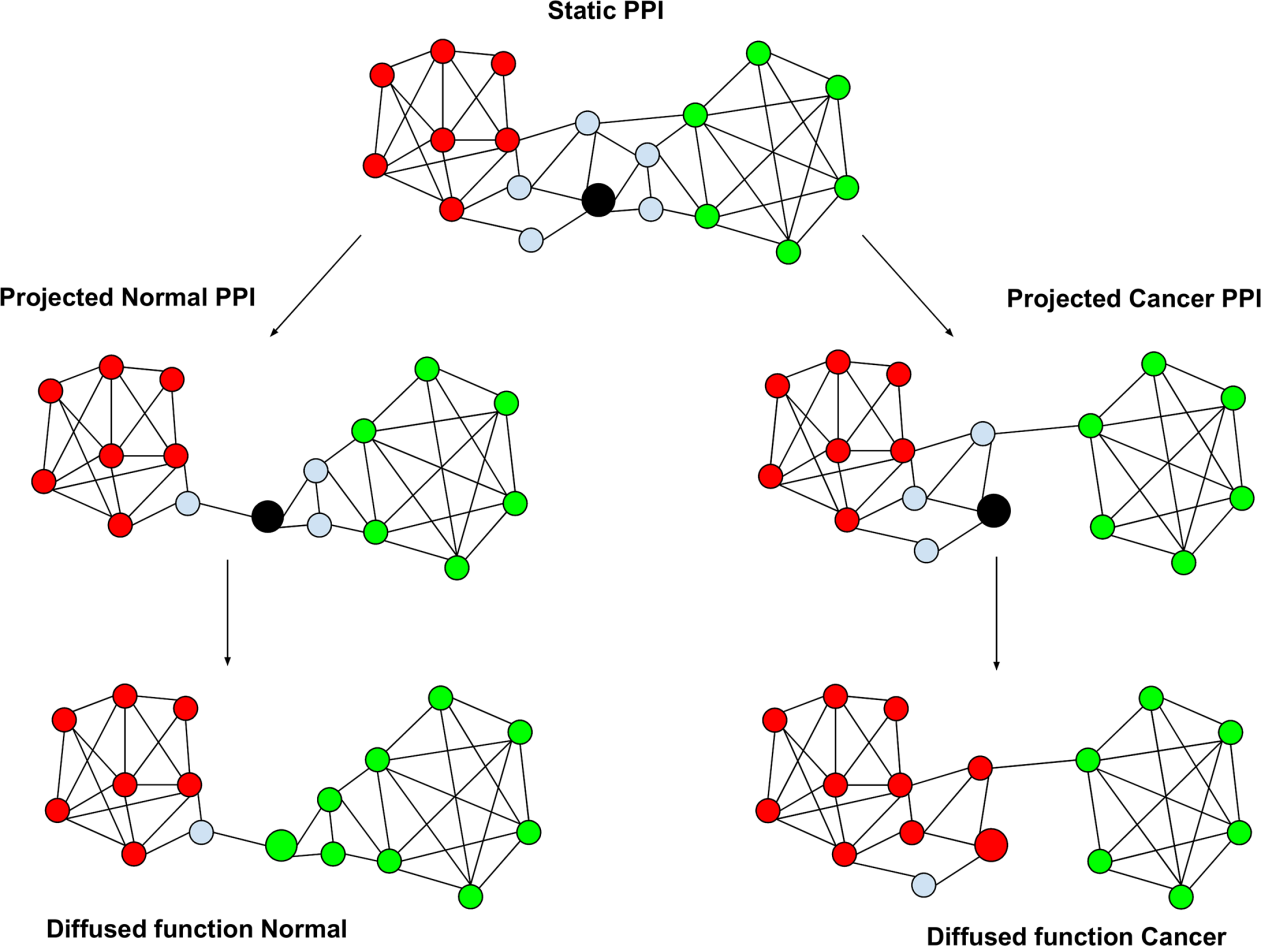
Overall approach. The reference gene is depicted by black circle. The initial static global PIN is projected onto normal and cancer samples based on gene expression, and each function (red and green) are diffused through each PIN. In this case, the reference gene is assigned green function in normal and red function in cancer, i.e., the gene gained red and lost the green function in cancer.

Our overall strategy is to (1) project PIN onto each transcriptomic sample, (2) diffuse functions across the sample-specific PIN to estimate sample-specific function of each gene, and (3) analyze functional changes across conditions (Fig 1). This pipeline specifically aims to uncover significant PPI alterations in the functional neighborhood of a gene which may potentially alter its function. Starting from a previously curated PIN [18], with 16,562 genes and 262,780 edges, we project the PIN on each sample-specific transcriptome, by removing the nodes corresponding to unexpressed or lowly expressed genes (RPKM < 1; see Methods), to obtain a sample-specific PIN. This general approach to obtain a sample-specific network has been successfully used before to obtain tissue-specific networks in human [20]. Furthermore, the notion of diffusion of information over sample-specific networks has previously been applied by Magger et. al [21] to identify tissue specific disease genes.

For each of the 1184 functional terms (1175 GO terms and 9 NetPath cancer-related pathways, see Methods), in each of the 1157 sample-specific PINs (110 breast cancer samples and 1047 normal breast tissue samples from TCGA[17]), we diffuse the functional annotation information across the network starting from *a priori* annotated genes in that network to yield a raw score for each node. The significance of the raw score is then estimated based on a null distribution of scores obtained by diffusing randomly annotated gene sets (Methods). For each function and sample, the set of genes with significant raw scores (p-value ≤ 0.01) are deemed to be involved in that function in the specific sample (red and green in Fig 1). Such sample-specific diffusion-based functional inference across normal and cancer samples allows us to identify specific genes that significantly gain or lose a function in cancer samples, and to assess whether a function has significantly gained or lost genes performing the function in cancer.

### Network diffusion reveals significant functional changes in breast cancer

After diffusing each of the 1184 functional terms across 110 normal and 1047 breast cancer samples, we assessed for each gene *g* whether the fraction of samples in which *g* is deemed to have the function is significantly different between the normal and tumor tissues based on a Fisher exact test; a greater fraction in cancer is referred to as *functional gain* and the opposite as *functional loss*. In addition to statistical significance, we require that the ratio of the fractions of samples where the gene is deemed to have the function in cancer versus normal ≥ θ (gain), or ≤ 1/θ (loss). The default value used in the main results is θ = 10, however, our conclusions are robust for θ from 2 to 10 (See Methods). We denote by *Δ*_*f*_ the difference between the number of genes deemed to have gained function *f* and the number of genes deemed to have lost it. Positive values of *Δ*_*f*_ indicate net gain and negative values indicate net loss of that function in cancer relative to normal. In total, 732 functions are predicted to undergo a net loss in cancer and 417 are predicted to have a net gain. Table 1 lists the top 10 functions gained and lost.

**Table 1:** Functions ranked based on functional variability of genes. Top 10 gained (green) and lost (red) functions are shown, along with *Δ*_*f*_, *Δ*_*f*_ divided (normalized) by the number of genes annotated by the function, followed by the sample shuffling, and the log fold change, which is the log ratio of the average number of expressed genes annotated by *f* in cancer and normal samples.

GO ID	Description	*Δ*_*f*_	Normalized *Δ*_*f*_	*Δ*_*f*_ (After Sample shuffling)	Normalized *Δ*_*f*_ (After Sample Shuffling)	log fold change
GO:0048661	*positive regulation of smooth muscle cell proliferation*	893	15.13	11	0.18	-0.04
GO:0048010	*vascular endothelial growth factor receptor signaling pathway*	744	10.19	-28	-0.38	-0.01
GO:0051279	*regulation of release of sequestered calcium ion into cytosol*	740	13.21	0	0	-0.03
GO:1901983	*regulation of protein acetylation*	723	12.05	-10	-0.16	-0.04
GO:0000910	*cytokinesis*	527	6.84	-8	-0.10	-0.02
GO:0010676	*positive regulation of cellular carbohydrate metabolic process*	523	8.43	-22	-0.35	-0.05
GO:0051291	*protein hetero oligomerization*	508	5.90	12	0.13	-0.03
GO:0042552	*myelination*	394	6.67	-6	-0.10	-0.03
GO:2000756	*regulation of peptidyl-lysine acetylation*	369	6.47	-10	-0.17	-0.03
GO:0016575	*histone deacetylation*	333	5.64	-17	-0.28	-0.01
GO:0006334	*nucleosome assembly*	-310	-3.13	-3	-0.03	0.04
GO:0051148	*negative regulation of muscle cell differentiation*	-127	-2.49	-15	-0.29	-0.06
GO:0007032	*endosome organization*	-75	-1.27	-2	-0.03	-0.007
GO:0018022	*peptidyl-lysine methylation*	-65	-0.91	-10	-0.14	0.002
GO:0007052	*mitotic spindle organization*	-64	-1.05	-15	-0.24	0.005
GO:0019886	*antigen processing and presentation of exogenous peptide antigen via MHC class II*	-56	-0.62	2	0.02	0.003
GO:0016236	*macroautophagy*	-53	-0.71	-13	-0.17	-0.01
GO:2000117	*negative regulation of cysteine-type endopeptidase activity*	-52	-0.61	-4	-0.04	0.005
GO:0051437	*pos reg of ubiquitin-protein ligase activity involved in regulation of mitotic cell cycle transition*	-51	-0.68	3	0.04	0.006
GO:0031145	*anaphase-promoting complex-dependent catabolic process*	-43	-0.57	7	0.09	0.006

Note that for a function if most genes annotated to have that function are differentially expressed between normal and tumor tissues, then *Δ*_*f*_ will simply reflect this differential expression of the annotated genes and not the effect of altered PIN. To ensure that our inference of functional loss and gain is independent of the genes annotated to have the function, when calculating *Δ*_*f*_, we exclude the genes annotated with the function. Moreover, in estimating functional gain and loss, we also exclude genes that are differentially expressed between normal and cancer, and consistently, as shown in Table 1, the functions inferred to have been lost or gained based on *Δ*_*f*_ exhibit modest log fold changes between normal and cancer, and therefore may go undetected based on differential expression based approach. As an alternative control approach, we rank functions based on expression differences of *a priori* annotated genes across normal and cancer. That is, the log fold change in number of expressed (≥1 RPKM) annotated genes across normal and cancer. Table 2 lists the top and bottom 10 of the 1184 functions ranked by average log fold change in number of expressed annotated genes. As seen in Table 2, only 2 of the functions ranked highly by our network based approach are also ranked highly by the expression based approach. Overall, we see a weak inverse correlation between *Δ*_*f*_ and the log fold change based on expression (Spearman rank correlation = -0.09). Thus, our approach uniquely reveals cancer-associated functions. For instance, we find *mitotic spindle organization* to be lost in cancer consistent with previous reports associating spindle misalignment with cancer [22]. Likewise, we find *positive regulation of smooth muscle cell proliferation* to be gained in cancer, consistent with prior studies [23]. The top 50 gained and lost functions are provided in S1 Table (S1 Table). As an additional control, we also compared the top functions ranked by our approach to the top functions ranked by the gene set enrichment analysis approach, further demonstrating the value added by our approach (See supplementary note S1 Text).

**Table 2:** Functions ranked based on expression variability of genes. Top 10 (green) and bottom 10 (red) functions are shown based on log fold change of expression based activity (that is, number of annotated genes present in the corresponding projected PIN), along with *Δ*_*f*_, *Δ*_*f*_ divided (normalized) by the total number of genes annotated by the function, followed by the sample shuffling results, and the log fold change.

GO ID	Description	*Δ*_*f*_	Normalized *Δ*_*f*_	*Δ*_*f*_ (Sample shuffling)	Normalized *Δ*_*f*_ (Sample Shuffling)	log fold change
GO:0006342	*chromatin silencing*	38	0.74	-1	-0.01	0.08
GO:0006334	*nucleosome assembly*	-310	-3.13	-3	-0.03	0.06
GO:0045814	negative regulation of gene expression, epigenetic	17	0.25	-3	-0.04	0.04
GO:0034728	*nucleosome organization*	4	0.03	-3	-0.02	0.03
GO:1990138	*neuron projection extension*	-10	-0.20	-7	-0.14	0.03
GO:0016458	gene silencing	7	0.04	-6	-0.03	0.03
GO:0031060	*regulation of histone methylation*	19	0.37	-15	-0.29	0.03
GO:0065004	*protein-DNA complex assembly*	22	0.14	-11	-0.07	0.03
GO:0031047	*gene silencing by RNA*	4	0.04	-7	-0.07	0.02
GO:0071824	*protein-DNA complex subunit organization*	8	0.04	-3	-0.01	0.02
GO:1901379	*regulation of potassium ion transmembrane transport*	14	0.25	-3	-0.05	-0.12
GO:0043266	*regulation of potassium ion transport*	10	0.12	1	0.01	-0.12
GO:1904064	*positive regulation of cation transmembrane transport*	309	5.15	0	0	-0.10
GO:0019229	*regulation of vasoconstriction*	206	3.32	-1	-0.01	-0.10
GO:0001508	*action potential*	2	0.03	1	0.01	-0.09
GO:0048871	multicellular organismal homeostasis	-9	-0.08	-2	-0.01	-0.09
GO:0051148	*negative regulation of muscle cell differentiation*	-127	-2.49	-15	-0.29	-0.09
GO:0034764	positive regulation of transmembrane transport	3	0.03	-7	-0.07	-0.09
GO:0034767	positive regulation of ion transmembrane transport	205	2.38	5	0.05	-0.09
GO:0050891	multicellular organismal water homeostasis	-31	-0.57	-5	-0.09	-0.09

### Predicted loss of function is supported by mutation and CNV profiles in breast cancer

Our analysis above identifies functions with net loss or net gain in cancer induced by PIN changes. For such a function *f*, if a gene *g* exhibits PIN-induced loss or gain of function *f*, then it is likely that mutation-induced loss or gain of *f* may also be linked to cancer. In other words, one might expect an elevated nonsense mutation (protein truncating) frequency among genes contributing to the loss of certain functions and elevated missense mutation frequency (amino-acid modifying) among genes contributing to the gain of certain functions. To this end, we assessed for each function if it exhibits an elevated nonsense mutation frequency among its lost genes and likewise, an elevated missense mutation frequency among its gained genes (Methods); we explicitly excluded the genes annotated with the specific function. We find that compared to lost functions, a greater fraction of gained functions exhibit an elevated missense mutation frequency (Fisher test odds ratio 2.13, p-value: 0.01). For robustness, we repeated this analysis for all settings of *θ* from 2 to 10 and additionally for *θ* = 2 combined with FDR < 0.1 to ascertain loss/gain of a gene relative to a function. In all 10 tests, we consistently observe a significant enrichment of elevated missense mutation frequency among gained functions, with an average odds ratio of 4.58. However, no such enrichment was observed for elevated nonsense mutation frequency among lost functions (average odds ratio = 0.94). As an alternative, we directly quantified Spearman correlation between Δ_*f*_ and mean missense mutation frequency of corresponding gained genes. Again, in all 10 cases, consistent with our expectation, we get an average spearman correlation of 0.28. Likewise, in all 10 test cases we find a weak inverse correlation with an average of -0.11 between mean nonsense mutation frequency of lost genes and Δ_*f*_.

Instead of mutations, when we use deletion CNV to quantify loss in activity (Methods), we find that compared to gained functions, a larger fraction of lost functions exhibited an elevated deletion CNV rate (Table 3). While the Fisher test p-value was marginal (0.09), the odds ratio was 2.15. After repeating the tests as above for other values of *θ*, in 8 of the 10 tests, the odds ratio > 1, with an average odds ratio of 1.59. As an alternative, we directly quantified Spearman correlation between Δ_*f*_ and deletion CNV rate of corresponding lost genes across all functions. In all 10 test cases, consistent with our expectation, we found a weak inverse correlation, -0.10 on average.

**Table 3:** Links between functional loss and mutation and deletion CNV. The Fisher test contingency table showing the distribution of functions with elevated missense mutation frequency (columns 2 and 3) and deletion CNV rates (columns 4 and 5) between lost and gained functions. *Mut(f) = 1* denotes significantly higher missense mutation frequency among the genes contributing to functional gain. *CNV(f) = 1* has an analogous interpretation for deletion CNV.

	*Mut(f) = 1*	*Mut(f) = 0*	*CNV(f) = 1*	*CNV(f) = 0*
***Δ***_***f***_ ***< 0***	23	709	26	706
***Δ***_***f***_ ***> 0***	27	390	7	410

Furthermore, we inspected the known mutation patterns of driver genes. Vogelstein et al [24] identify 125 pan cancer driver genes, 71 of which are classified as tumor suppressors and 54 as oncogenes based on their recorded pattern of missense and nonsense mutations in the COSMIC database [25]. We filter this set to only consider known breast cancer drivers. That leaves us with a total of 51 genes (39 tumor suppressors and 12 oncogenes). One would expect genes with mutation patterns of tumor suppressors to lose functions, while genes with mutation pattern of oncogenes to gain functions. Hence, we test whether the tendency of a gene to gain function (respectively lose function) is an indicator of its oncogene status (respectively tumor suppressor status) from its ROC-AUC value. The tendency of each gene to gain (respectively lose) function is the fraction of all 1184 functions predicted to have been gained (respectively lost) by that gene. As a control, we generate a null distribution of 2000 AUC values using random labelling of oncogenes (respectively tumor suppressors) and generate empirical p-values. We get a strong association signal when we test if the 12 breast oncogene drivers (AKT1, BRAF, EGFR, ERBB2, FLT3, IDH1, KLF4, KRAS, MED12, NRAS, PIK3CA and SF3B1) have a higher tendency to gain functions relative to the rest (AUC-ROC value = 0.737, p-value: 0.0025) whereas the 39 breast tumor suppressor drivers did not have a higher tendency to lose functions relative to the rest (AUC-ROC value = 0.51, p-value: 0.405). We additionally checked if there is signal in the opposite direction, that is, whether breast oncogenes (respectively tumor suppressors) have a higher tendency to lose (respectively gain) functions than the rest, and did not detect a significant signal (AUC-ROC p-value for oncogenes: 0.301, AUC-ROC p-value for tumor suppressor: 0.245). For robustness, we repeat the analysis under different settings of gain/loss threshold *θ* and additionally for *θ* = 2, FDR < 0.1. Consistently, in all 10 cases, there is a strong association signal between oncogene status and tendency to gain function (Average AUC = 0.74). With respect to tumor suppressors, across all 10 tests, we consistently observe a lack of association between tumor suppressor status and tendency to lose function (average AUC = 0.53). These results are consistent with several reports implicating somatic gain of function mutations in oncogenes such as PIK3CA and KRAS and tumor suppressor genes such as TP53 [26][27][28]. Overall, these results suggest that a change in network neighborhood of a gene may provide an alternative mechanism for functional loss, in addition to mutations and deletion CNVs.

### Predicted direction of change in a function’s activity is associated with its effect on patient survival

We further assessed whether functions that exhibit cancer-associated gain or loss also exhibit a consistent association with patient survival. For instance, for a function with net loss in cancer relative to normal tissues, we expect that among cancer patients the lower the activity of the function, the worst the patient survival (and the converse for gained functions). To test this association, for each function we estimate its sample-specific activity as the number of genes inferred to be performing that function based on diffusion scaled across all samples. We then estimate the association between patient survival risk and our diffusion-based sample-specific activity of each function using a Cox proportional hazard regression model adjusted for differences in age, and stratified by sex and race. A significant negative (respectively, positive) regression coefficient β corresponds to negative (respectively, positive) association with risk. Of the 1149 functions (732 net loss and 417 net gain), 137 exhibited significant association with survival risk (p-value ≤ 0.05). Of these, 111 were negatively associated with risk, and interestingly, these were significantly biased toward lost functions, consistent with our hypothesis (Table 3, columns 2 and 3; Fisher test p-value = 1.1E-3; odds ratio = 2.1). Only 26 of the 137 were positively associated with risk, but consistently, these were biased toward gained functions (Table 4, columns 4 and 5; Fisher test p-value = 5.1E-5; odds ratio = 5.7). As an alternative assessment, we found a positive correlation between Δ_*f*_ and *β* (Spearman correlation = 0.29). These results suggest that diffusion-based inference of cancer-associated functional change may also be associated with the severity of the tumor among cancer patients. We repeated the above analyses for all values of *θ* from 2 to 10 and additionally for *θ* = 2 combined with FDR < 0.1 to ascertain loss/gain of a gene relative to a function. As shown in S3 Table (S3 Table), 29 of the 30 tests are consistent with the results above.

**Table 4:** Change in functional activity and association with patient survival. Fisher test contingency table to test for association between functional loss/gain with associations with patient survival; β indicates the association of tumor-specific functional activity with survival risk.

	β<0 & p-value ≤ 0.05	p-value > 0.05	β>0 & p-value ≤ 0.05	p-value > 0.05
***Δ***_***f***_ ***< 0***	87	639	6	639
***Δ***_***f***_ ***> 0***	24	373	20	373

### Diffusion-based functional activity profile of a tumor is predictive of patient survival

Encouraged by the results above, we directly assessed the power of our diffusion-based sample-specific activity profile of a function in predicting patient survival. To this end, we selected the top 1% and bottom 1% (24) most cancer-associated functions (ordered by ***Δ***_***f***_), and for each function we estimated its diffusion-based activity in each tumor sample, as defined above. Using the inferred activity levels of these 24 functions as sample-specific features, we then computed the cross-validation accuracy of patient survival prediction based on multivariate Cox regression (Methods). The prediction accuracy was quantified using the standard concordance or C-index metric [29]. We find that cross-validation C-index is 0.567. As a control, we assessed whether the alternative approach to quantify sample-specific functional activity, based simply on number of annotated genes in each sample-specific network could be equally effective. For candidate features, we assessed the median number of annotated genes in each sample-specific network and identified 24 most differentially active functions based on the absolute log ratio of the medians in cancer and normal samples. We then quantified sample-specific activity of these 24 functions based on the number of annotated genes in each sample-specific network scaled across all samples and estimated the concordance in an identical fashion to our diffusion-based approach above. This yielded a C-index of 0.55, which is not significantly worse. However, it still demonstrates the complementary value of our approach. We further included the 9 known cancer-signaling pathways from the NetPath database [30], namely, *EGFR1*, *FSH*, *IL-1*, *IL-4*, *IL-5*, *Leptin*, *RANKL*, *TNF-alpha*, *and TSH*. On using the diffusion based profiles of this extended feature set of 33 functions, we get a cross-validation C-index of 0.62. It is interesting to note that although none of these pathways are ranked among the top cancer-associated functions based on diffusion, their addition nonetheless improved the model’s predictions. Additionally, we observe that the accuracy of the annotation based model reduces when including these 9 pathways as features (C index = 0.51) and it is significantly lower than the model using diffusion profiles (p-value 0.01). For robustness, we repeated this analysis for all values of *θ* from 2 to 10 and *θ* = 2; FDR < 0.1 which help determine 24 of the 33 features. As shown in S4 Table (S4 Table), excluding 1 case, the diffusion-based C-index is higher than the control, and significantly so in 8 of the cases when pathway information is incorporated.

We further validated the survival prediction accuracy of our diffusion-based functional activity profile in an independent METABRIC breast cancer dataset [12]. We used the two sets of 24 features derived from TCGA dataset as above and used those to assess cross-validation prediction accuracy of the diffusion-based and annotation-based methods in METABRIC. Here, we find that C-index of the diffusion-based approach was 0.615 whereas the annotation-based approach had a significantly lower C-index of 0.557 (difference p-value = 5.64E-05). We also recomputed the prediction accuracies after incorporating the 9 pathway profiles into the model. The C-index of the diffusion based approach was 0.62 whereas the annotation-based profiles achieved an accuracy of 0.57 (difference p-value = 0.0004). These consistent results across datasets suggest that the diffusion-based approach to quantify functional activity may provide additional information about the functional state of a tumor, relevant to patient survival.

### Diffusion-based functional activity profile of a tumor predicts clinical subtypes of breast cancer

Using the Δ_*f*_ derived features from TCGA, we further tested if our novel diffusion-based functional activity profile is predictive of known clinical characteristics of breast tumors, specifcially, the cancer subtype (Basal, Her2, Luminal A, Luminal B, Normal-like), and its hormone response status, Estrogen Receptor positive (ER+) and Progesterone Receptor positive (PR+). Based on clinical annotation of the METABRIC tumors, we trained 7 different Support Vector Machine (SVM) models, one per clinical indicator, using randomly selected 50% of the samples to train and the other half to assess the prediction accuracy, quantified by ROC-AUC. We repeated the training and testing 2000 times to obtain mean and 95% confidence interval. Note that while the training and testing of the model is done on METABRIC, the cancer-associated functions used as features were inferred from TCGA data independently as shown above. We compared the performance of our diffusion-based functional activity profile with annotation-based activity profiles as above. Table 4 shows the AUC estimates of each model. As shown in Table 5, we found in almost all classification tasks, the diffusion-based approach can predict each clinical indicator more accurately than the alternative annotation-based approach (one-sided Wilcoxon rank sum test all p-values < 2.2e-16). We additionally recompute the subtype prediction accuracies in the presence of the 9 pathways (See S5 Table) to find an overall improvement in prediction accuracies of both methods for some subtypes. However, on comparison, the diffusion based approach remains superior (all p-values < 2.2e-16).

**Table 5:** Prediction accuracues using diffusion based fuctional profiles and annotation based functional profiles quantified by AUC-ROC. The following table displays the AUC estimates of the 7 independent classifiers trained with two different feature sets (diffusion-based functional activity and annotation-based functional activity) for each clinical indicator.

Clinical Indicator	AUC—Diffusion	AUC–Annotation (Control)	P-value (AUC-Diffusion > AUC-Control)
*Basal*	0.87 (95% CI = 0.867–0.882)	0.81 (95% CI = 0.803–0.823)	<2.2e-16
*Her2*	0.73 (95% CI = 0.72–0.746)	0.65 (95% CI = 0.644–0.67)	<2.2e-16
*Luminal A*	0.74 (95% CI = 0.741–0.756)	0.73 (95% CI = 0.722–0.739)	<2.2e-16
*Luminal B*	0.73 (95% CI = 0.727–0.742)	0.75 (95% CI = 0.731–0.748)	1
*Normal*	0. 87(95% CI = 0.866–0.882)	0.81 (95% CI = 0.803–0.823)	<2.2e-16
*ER+*	0.88 (95% CI = 0.882–0.895)	0.81 (95% CI = 0.811–0.828)	<2.2e-16
*PR+*	0.74 (95% CI = 0.736–0.75)	0.72 (95% CI = 0.719–0.735)	<2.2e-16

**Fig 2:**
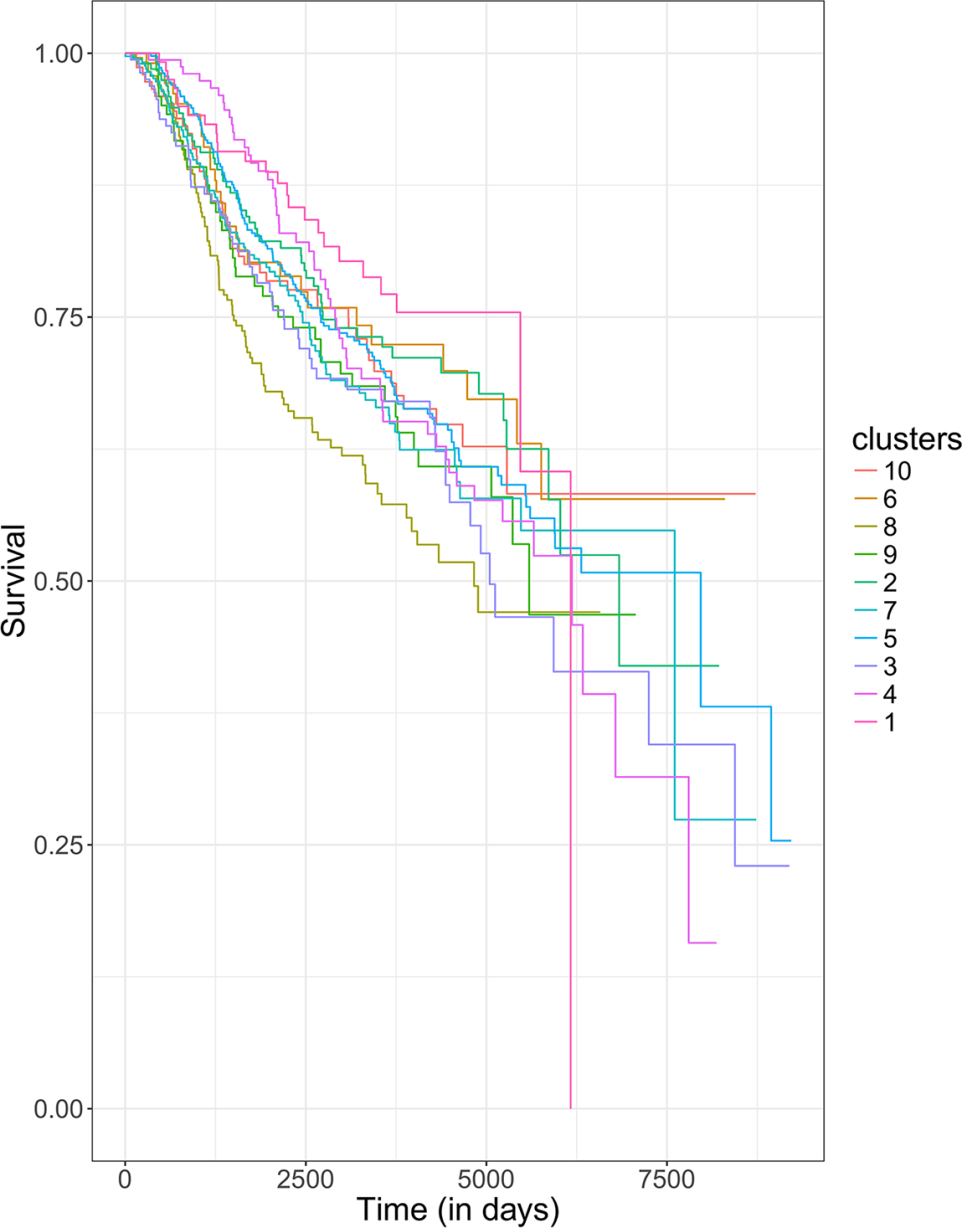
Clustering breast cancer samples based on their functional activity profile. Kaplan-Meier survival curves of patients grouped in the 10 clusters show significant survival differences.

Encouraged by the results above, we further investigated the diffusion based activity profiles of all the 33 functions (24 GO terms and 9 cancer-related NetPath pathways) used above. Specifically, we clustered all METABRIC samples using Nonnegative Matrix Factorization (NMF) [31], in an unsupervised fashion, into 10 groups (Methods). We found that the functional profile-based clustering are not associated with known histopathological subtypes of breast cancer. However, as seen in Fig 2, the diffusion-based unsupervised clusters exhibit significant inter-cluster differences in patient survival (Log rank p-value = 3.2E-3; Fig 2). In contrast, when we use annotation-based functional activity profiles to cluster the tumors following an identical procedure as above, the clusters did not reveal a difference in survival across clusters (Log-rank p-value = 0.23). Moreover, we fitted a Cox proportional hazards model to the METABRIC survival data using cluster membership as a feature while controlling for age, sex and race, as above. Cluster memberships generated by diffusion-based functional activity profiles show a significant association with survival risk (β = 0.04, p-value = 8.5E-3) whereas cluster memberships generated by annotation-based profiles had no significant effect (β = 0.01, p-value = 0.32). These results suggest that in addition to expression based changes, PIN-induced functional changes of genes in breast tumors may also play a functional role in cancer.

**Fig 3:**
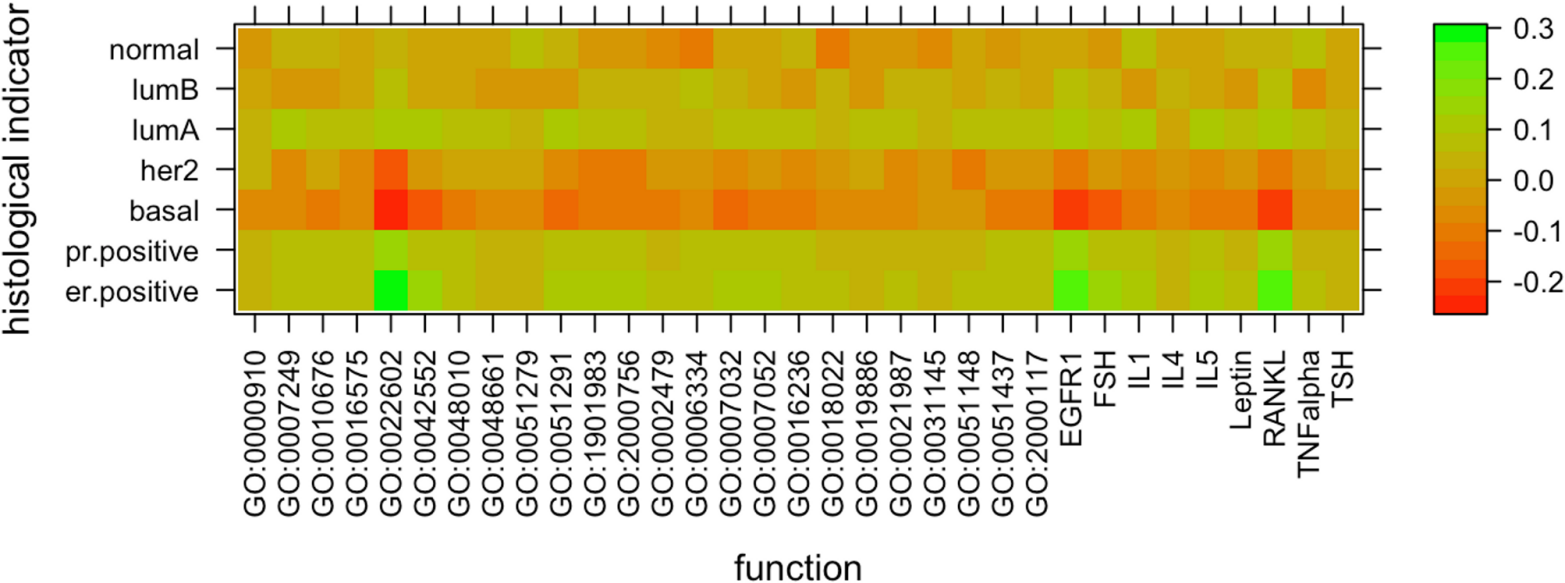
Diffusion based functional heterogeneity across clinical subtypes. The following figure displays the log ratio between the average numbers of genes assigned to each function by diffusion (represented by columns) across samples annotated with a subtype (represented by rows) versus the rest of the samples.

Fig 3 shows for each of 7 subtypes, the log-fold change in average diffusion based functional activity of the 33 functions (24 GO processes and 9 Netpath pathways) in samples corresponding to the subtype versus the rest. The most notable changes are increase in activity of *ovulation cycle process (*GO:0022602), *Epidermal Growth Factor Receptor signalling pathway (*EGFR1), and *Receptor Activator of Nuclear factor Kappa-B Ligand signalling pathway (*RANKL) in ER+ breast tumors. Previous experimental and clinical studies have indicated that EGFR based signalling in ER+ breast tumors leads to resistance to hormone therapy [32][33] through hormone independent proliferation of tumors [34]. As seen in Fig 3, the EGFR1 signalling pathway has a 0.23 log fold higher average functional activity in ER+ breast cancer patients (~70% of which were recorded to have taken hormone therapy).

Our results also indicate a 0.24 log fold higher functional activity of RANKL signaling pathway in ER+ breast cancer. One of the downstream outcomes of this signalling pathway is positive regulation of TNFRSF11A gene which encodes for RANK [35]. RANKL has been experimentally shown to induce cell migration in epithelial tumor cells expressing RANK, and is also an important osteoclast differentiation factor expressed in the bone marrow thereby creating a conducive environment for bone specific metastasis of RANK expressing tumor cells [36]. This is consistent with the observation that many tumors in breast that are known to recur in bone tissue are ER+ [37]. Moreover, inhibition of RANKL in combination with hormone therapy has been shown to improve treatment efficacy and prevention of bone metastasis in experimental mouse models of ER+ tumors [38]. These results suggest that the knowledge of PIN guided functional changes in genes via guilt by association may provide potential clues into yet unknown mechanisms of acquiring treatment resistance.

## Discussion

Traditional approaches to assess functional changes in a specific condition rely entirely on a priori functional annotation of genes and quantify the differential expression levels of genes a priori known to perform a specific function [39]. However, current functional annotations are highly incomplete, and importantly, lack contextual information. We have presented a novel method based on network diffusion that leverages sample-specific PIN [20,40–42] to assess sample-specific function of a gene, thus refining and extending the prior annotations by applying the principle of guilt-by-association. This offers a complementary approach to identify genes that have gained or lost a specific function, or broadly, identify functions that are gained or lost, in cancer relative to normal tissues. Moreover, this is the first application of network diffusion to infer sample-specific gene function and using them to identify functional changes associated with cancer. We have shown the efficacy of our approach in uniquely detecting cancer-associated functions, as well as providing a complementary functional characterization of a tumor sample and compared with traditional approach relying on functional annotations alone, our approach is more predictive of patient survival, as well as known histopathological subtypes of breast cancer.

Two previous methods–DECODE [43] and MetaDCN [44] were used to identify cancer-associated functions. Our method has some key differences from these previous approaches. First, both DECODE and MetaDCN rely on gene expression or co-expression variability to reveal cancer associated functions. Hence, in the context of cancer vs normal, these approaches would not identify potentially important mutated functional gene sets whose constituent genes do not vary substantially in their expression levels across all samples. As shown, despite controlling for expression variability, our approach captures important functional gene sets, some of which are enriched for nonsynonymous mutations. Second, more fundamentally, our approach leverages a gene’s potential functional variability explicitly due to changes in context-specific PPI whereas differential co-expression methods leverage changes in correlated expression, which assumes certain transcriptional regulatory mechanisms. Third, our approach can uniquely be applied to extract functional gene sets at the sample-specific level.

Our diffusion-based approach to functional assignment has a few limitations. First, the guilt-by-association is a trend and there are several exceptions to the general principle, as noted previously [45], and second, the diffusion is effective for relatively large functional groups. We have explicitly addressed these limitations by restricting our analysis to those functional groups that yield a reasonable diffusion-based recall (Methods), suggesting that these functions are broadly clustered in the PIN, and by only considering functional groups with at least 50 genes (and at most 500 genes, as discussed in Methods). It is interesting to note that the number of genes implicated in a function can far exceed the number of genes currently annotated by the function, consistent with substantive incompleteness of functional annotations. However, it is difficult to verify these predicted functional implications, except indirectly through their predictive value in various tasks, as we have done. Third, our approach only relies on gene expression-based inference of inactivation to generate sample-specific networks. While it would be informative to use other data sources to inform the sample specific network, one would need to carefully account for potential biases that may arise. For instance, mutation and CNV data can be incorporated in the sample-specific network generation process. However, it is not clear if a nonsense mutation will inactivate the gene product. Hence, previous studies [46][47] have also used expression-based filtering and accordingly in this initial work we refrain from incorporating additional data to generate sample specific networks. Additionally, our sample-specific network generation process is less flexible compared to alternative described in [21] that employ edge re-weighting to penalize edge weights based on expression rather than completely removing nodes. However, their approach requires an additional re-weighting parameter that needs to be pre-determined. It would be interesting to see if edge re-weighting further improves our results. Fourth, although we have applied symmetric normalization of edge weights by square root of product of node degrees in the PPI networks to control for degree bias in diffusion, we expect that additionally controlling for degree bias by performing degree-aware sampling of random seeds as described in DADA [48] might further help refine our predictions.

In the particular breast cancer application of our approach presented here, a few follow up investigations will provide further insights and strengthen our conclusions. For instance, it will be instructive to focus on specific genes that contribute to functional gain of important oncogenic functions. These genes may provide alternative therapeutic targets.

In sum, our work suggests a novel framework to investigate dynamic changes in a gene’s function through diffusion of a function in the context-specific PIN. While our initial application was to investigate functional changes in breast cancer in human, the methodology is equally applicable to other organisms and other dynamic contexts such as other diseases, development, and tissues.

## Methods

### Breast cancer data

From The Cancer Genome Atlas (TCGA)[17] we obtained: (i) RNASeq data for a cohort of 110 normal breast samples and 1047 breast cancer samples, (ii) the somatic mutational status of each gene for each breast cancer sample (mutations are called relative to matched normal sample), (iii) the mean normalized segmental copy number variation (CNV) value relative to the reference genome (*log*_*2*_ ratio), and (iv) the survival data for the patients corresponding to the breast cancer samples. We estimated the somatic mutation and CNV frequency of each gene in breast cancer samples. The missense (respectively, nonsense) mutation frequency of a gene was defined as the fraction of samples where that gene has at least 1 missense (respectively nonsense) mutation. For CNV frequency, chromosome segments were mapped to overlapping genes to estimate the normalized somatic CNV profile for each gene across breast cancer samples using GISTIC version 2.0 [49]. Based on this profile, we estimated the CNV amplification (respectively, deletion) frequency of each gene as the fraction of samples where the amplitude of variation is > *log*_2_(1.2) (respectively, < *log*_2_(1/1.2)). For independent validation, from METABRIC [12], we obtained: (i) Microarray data for a cohort of 1989 breast cancer samples (ii) clinical and survival data corresponding to each cancer sample.

### Protein interaction network (PIN)

We used the HIPPIE (version 2.0)[18] PIN, which integrates multiple human protein interaction datasets and provides a confidence score for each interaction. Overall, the HIPPIE PIN consists of 16,562 genes and 262,780 scored physical interactions. For each sample, we consider a gene as *expressed* if its expression is ≥1 RPKM (Reads per Kilobase of transcript per Million mapped reads) in that sample. This threshold roughly corresponds to 1 mRNA per cell and in previous studies has been shown to yield negligible false discovery rate at ~20% false negative rate [50]. We obtained a sample-specific PIN by retaining only the expressed genes in that sample. The average number of nodes in the PPI networks across all samples is ~ 13,019 and the average number of edges is ~204,506. Additionally, on average across all tumor samples we observe 164 connected components with 442 out of the 1184 functions considered, spanning more than 1 connected component. Nonetheless, our network propagation technique is still likely to find a solution due to the bounded Eigen values of the propagation matrix. The sample-specific network statistics for tumor samples in provided in S2 Table. To maintain consistency with the METABRIC gene expression dataset, which was generated on a microarray platform, we accordingly use a threshold whose percentile value equals that of 1 RPKM in the TCGA dataset.

### Network diffusion algorithm for sample-specific functional assignment to a gene

Let *G*(*V*, *E*) be the weighted undirected network with *V* representing the set of nodes and *E* the set of weighted interactions. *Let W* be the weighted adjacency matrix corresponding to *G* and let *D* be the diagonal degree matrix (with diagonal entries corresponding to the weighted degree of each node in the graph). For a function *f*, let *A*_*f*_ be the set of genes annotated with that function. For a RNA-seq sample *s*, given the sample-specific graph *G*_*s*_(*V*_*s*_, *E*_*s*_), let *Y*_*s*_ be the prior knowledge vector such that *Y*_*s*,*g*_ = 1 *if g* ∈ *A*_*f*_ ∩ *V*_*s*_. The guilt-by-association principle implies that the involvement of any gene *g* in a function is likely to be similar to those of the genes in its neighborhood. Additionally, the involvement should be consistent with our prior knowledge of functional activity. This can be mathematically modelled by the following diffusion equation:

$$F_{s}=(1-\alpha )(D_{s}^{-\frac{1}{2}}W_{s}D_{s}^{-\frac{1}{2}})F_{s}+\alpha Y_{s}$$ (1)

Here *F*_*s*_ is a vector of raw involvement scores of every gene in *G*_*s*_ *α* ∈ (0,1) is a parameter that weighs the importance of prior knowledge in the model. Notice that the adjacency matrix *W*_*s*_ is symmetrically normalized by the square root of the product of node degrees. This step controls for biases that may arise from diffusing information to high degree nodes (hubs) in the network. As shown in previous work, the raw scores are robust for the choice of α, and we adopt the choice α = 0.2 following (Vanunu et al., 2010). Since the Eigenvalues of $D_{s}^{-\frac{1}{2}}W_{s}D_{s}^{-\frac{1}{2}}$ lie in [–1,1], it can be shown that $I-(1-\alpha )D_{s}^{-1/2}W_{s}D_{s}^{-1/2}$ is not singular and there exists a unique solution:

$$F_{s}=\alpha {\left(I-(1-\alpha )D_{s}^{-\frac{1}{2}}W_{s}D_{s}^{-\frac{1}{2}}\right)}^{-1}Y_{s}$$ (2)

This solution can be efficiently computed using the general iterative matrix multiplication algorithm first proposed by Zhou [51]. Since the system is also symmetric and positive definite, we instead obtained our solution using the conjugate gradient (CG) method already implemented in the C++ Eigen library.

The above procedure assigns a raw involvement *score* to each gene in *G*_*s*_ for each diffused function. The raw score however depends on |*A*_*f*_ ∩ *V*_*s*_| as well as the sample-specific PIN topology. To appropriately calibrate it, we can estimate a significance p-value for the score, in a function-specific manner. This is done by comparing a gene’s raw score against a null distribution of scores generated by diffusing random prior knowledge vectors in *G*_*s*_ annotating |*A*_*f*_ ∩ *V*_*s*_| genes. Hence each null distribution is parameterized by |*A*_*f*_ ∩ *V*_*s*_| which we call the *seed size*. Note that this technique requires us to run a large number of bootstrap instances separately for each sample-specific PIN (1157 samples in total) for each function (1184 in total). Nonetheless, to make the computation feasible, we follow a memoization procedure in which for each sample, we partition the set of all functions into a fixed number of bins. Each bin has a pre-determined seed size given by the median seed size of all functions in that bin. The number of bins per sample are chosen such that each function with seed size *k* in a sample is assigned a bin with seed size at most *k* ± 10. A null distribution of raw scores is pre-computed for each bin in each sample by diffusing random prior vectors having the corresponding bin’s seed size in the projected network for that sample. We estimate p-values of diffused raw scores of a function in each sample by comparing them to the pre-computed null distribution of the bin it was assigned to in that sample. To save on additional overhead costs of storing and reading many large pre-computed tables, the null distributions were approximated as normal distributions with mean and standard deviation estimated by the sample mean and standard deviations from 100 bootstrap samples. Further information is provided in the supplementary note (See S1 Text). Finally, we say that a gene is *assigned* a function *f* in each sample if the p-value associated with its raw score in that sample < 0.01. We chose a relatively inclusive p-value threshold because our estimates are based on 100 bootstrap samples. However, a more stringent p-value of 0.001 results in highly consistent activity profiles for each function across samples (See S1 Fig).

### Functional annotation data

We extracted function annotations corresponding to human GO [52] biological processes and Netpath cancer and immunological pathways [30]. Although our approach is in principle, applicable to any function, factors such as inadequate data and pre-defined model assumptions may make it unsuitable for some functions in practice. Hence we apply 3 filtering criteria to exclude such functions. One issue with the diffusion of functional information from a small number of initially annotated genes is that it results in a statistically weak calibration of the raw score. On the other hand, broadly annotated functions suffer from the difficulty of biological interpretation. Hence, as the first criterion, we restrict ourselves to GO terms and pathways having 50 to 500 annotated genes. Second, since the PIN is still inevitably incomplete, diffusion of functional information from annotated genes that are poorly connected to the rest of the PIN (due to possibly missing or low confidence edges) is less likely to be sensitive to network changes across samples, and are therefore are not suitable. Hence as the second criterion, we assessed for each function whether it adheres to guilt-by-association principle, by estimating the average recall value of each function (fraction of all genes annotated by that function that were successfully re-assigned this function by diffusion over the general network when performed in a leave-one-out manner). We only retained functions with an average recall value ≥ 0.1. Finally, as the third filtering criterion, we required that the functions be modestly active in breast tissue. Hence, for each sample, we measured the fraction of genes annotated with *f* that were expressed (RPKM ≥ 1) in that sample. Overall, we observed that the average value of this fraction across all functions passing the first two criteria, and over all samples, was ~0.85. We retained a function for further analysis only if it had this mean value (0.85) in at least 20% of all samples. This only excludes the least relevant functions, and overall, after applying these filters, 1175 GO biological processes and 9 Netpath cancer signaling pathways were retained for further analyses.

### Quantifying cancer-associated loss and gain of a gene relative to function

Given a cohort of samples under two conditions (normal and breast cancer in our application) and a gene-function pair *(g*,*f)*, we determine the number of samples where *g* was assigned function *f* by diffusion (see above). Having determined this separately for normal and breast cancer samples, we perform a Fisher’s exact test to assess whether the assignment of *f* to *g* is significantly enriched in either one of the conditions. We say that a gene *g* lost a function *f* in cancer if the assignment of *f* to *g* is significantly enriched among normal samples when compared to cancer. Conversely, a gene *g* gains a function *f* in cancer if the assignment of *f* to *g* is significantly enriched among cancer samples when compared to normal. The enrichment P value is determined by Fisher exact test based on the contingency table (Table 6). Unless stated otherwise, we use the default p-value significance threshold of 0.05.

$$\mathrm{Odds}\;\mathrm{ratio}\;(\theta )=\frac{n_{00}\times n_{11}}{n_{10}\times n_{01}}$$ (3)

**Table 6:** Contingency table. The table generated after performing diffusion based function assignment of a function to gene g in each tumor and normal sample.

(*g*, *f*)	Assigned	Not assigned	Total
Cancer	*n*_00_	*n*_01_	= (*n*_00_ + *n*_01_)
Normal	*n*_10_	*n*_11_	= (*n*_10_ + *n*_11_)
Total	= (*n*_00_ + *n*_10_)	(*n*_01_ + *n*_11_)	*n* = (*n*_00_ + *n*_01_ + *n*_10_ + *n*_10_)

Although the results presented here are under the nominal significance setting of p-value < 0.05, we additionally estimate the false discovery rate under this setting and discuss the robustness of results after correcting the p-values for the number of genes tested. Further explanation is provided in the supplementary note (See S1 Text). In addition to the significance criteria, we also consider the effect size of the functional gain or loss. θ is the odds ratio derived from the Fisher contingency table. We require the effect size to be large; for various analyses, we used a range of *θ* from 2 to 10, and unless otherwise mentioned, the default is highly stringent θ = 10, while the results for other values of θ are provided in the Supplementary material. Note that if a gene is not expressed in a sample then it is not present in the sample-specific PIN and therefore cannot be assigned a function. Thus, if *g* is un-annotated by a function, biases may arise in the determination of its gain or loss if there are significant differences in the expression of *g* in sample-specific networks generated within a cohort or between two cohorts. To control for such a bias, we take two filtering measures. First, we check if *g* is expressed significantly more in samples corresponding to one condition relative to the other by building a contingency table for expressed versus not expressed among normal and cancer samples and performing a Fisher exact test. We exclude *g* if its p-value ≤ 0.05. Second, in estimating loss and gain for *g* relative to a function we only consider samples where *g* was expressed in the sample. This results in further downstream analyses of 12599 genes out of a total of 16562 from the original network.

### Quantifying cancer-associated loss and gain of a function

Below, in assessing loss or gain of a function in cancer relative to normal, we only consider the genes that are not annotated to have that function. This ensures that our estimated change in functional activity is informed primarily by the changes in PIN topology and not by the differential expression of the genes annotated to perform a certain function. Define

$$\begin{array}{l} Ø(f,g)=1\;if\;g\;is\;un\text{-}annotated\;and\;gains\;f\\ \;=\text{-}1\;if\;g\;is\;un\text{-}annotated\;and\;loses\;f\\ \;=0\;otherwise \end{array}$$

Let Δ_*f*_ = ∑_*g*_ *ϕ*(*f*, *g*) be the difference between the number of un-annotated genes gaining and losing *f*. The higher the |Δ_*f*_| value, the greater the change in activity of *f* between normal and cancer. The direction of change is determined by the sign: “+” represents increase in activity from normal to cancer due to a greater number of un-annotated genes potentially acquiring that function in cancer; we refer to such a function as *cancer-associated gained function*. Likewise, “-” represents an overall decrease in functional activity due to a greater number of un-annotated genes potentially losing that function in cancer; we refer to such a function as *cancer-associated lost function*.

### Assessing whether a gene set has elevated mutation frequency and deletion CNV in cancer

For a given function *f*, we identify the set of unannotated genes that have lost the function in cancer for threshold θ ≤ 0.1 (respectively, gained f at θ ≥ 10). We then test if f exhibits an *elevated* nonsense (respectively, missense) mutation frequency by comparing the nonsense mutation (respectively, missense) frequency of the set to that of the rest of the genes using Wilcoxon test; define *Mut(f)* = 1 if Wilcoxon p-value ≤ 0.05 and *Mut(f)* = 0, otherwise. Next, to test whether cancer-associated lost functions have a greater tendency to exhibit elevated nonsense mutation frequency compared to gained functions, we perform a Fisher’s exact test on the following contingency table (Table 7):

**Table 7:** Contingency table. The following table is generated to determine if elevated missense (respectively nonsense) mutation frequencies are enriched among functions with net gain (respectively net loss).

	*Mut(f) = 1*	*Mut(f) = 0*
**Δ_*f*_ <** 0		
**Δ_*f*_ >** 0		

We perform analogous tests to assess whether cancer-associated lost functions have a greater tendency to exhibit elevated deletion CNV frequency compared to gained functions and whether cancer-associated gained functions have a greater tendency to exhibit elevated missense mutation frequency compared to lost functions.

### Patient survival prediction based on sample-specific functional activity

Given a sample *s* and a function *f*, let *X*_*s*,*f*_ be the number of genes assigned with function *f* in *G*_*s*_ by diffusion and let *X’*_*s*,*f*_ be the number of genes assigned with function *f* in *G*_*s*_ by annotation, i.e., $X_{s,f}^{\prime }=|A_{f}\cap V_{s}|$. We obtain a diffusion based functional activity profile *X*_*f*_ and an annotation based functional activity profile *X’*_*f*_ for each function across all samples. Let *F*_*k*_ be the set of top & bottom *k*% of functions ordered by *Δ*_*f*_ (respectively $F_{k}^{\prime }$, the set of top |*F*_*k*_| functions ordered by log fold change of median number of annotated genes across normal and cancer PINs). We estimate the patients’ survival risk using a multivariate Cox proportional hazards model when fitted in a 10-fold cross validated manner using *F*_*k*_ as the set of features and *X*_*f*_ normalized across all breast cancer samples as the sample-specific feature values (respectively for $F_{k}^{\prime }$ and $X_{f}^{\prime }$). The model is controlled for age and stratified by sex and race. Then, we estimate the concordance-index (*C-index) C* (respectively, *C*′) of the predicted risk-score and standard error *σ*_*C*_ (respectively, $\sigma_{C^{\prime }}$) following [29], based on feature values *X*_*f*_ (respectively, *X’*_*f*_), relative to the corresponding survival data. Based on the estimated standard errors and predicted risk scores of the two models on the same set of samples, we determine if *C* is significantly greater than *C*′ by deriving a t-statistic and its associated p-value following [53]. R *survival* and *survcomp* packages were used for these analyses.

### Clustering of diffusion-based functional activity profiles

Given a set of functions with their diffusion-based functional activity profiles, we use the Non-negative Matrix Factorization method [31] for clustering. Given a desired number of clusters *k* as input, we measure the quality of the clustering following the Dunn index metric [54] (ratio of the maximum inter-cluster distance to the minimum intra-cluster distance) normalized by number of clusters. For *k* ranging from 2 to 25, we compute the Dunn index normalized by *k* and plot the results (See S2 Fig). For *k* ≥ 10 there are no significant improvements, hence we stick to a choice of *k* = 10.

## Supporting information

S1 Text Supplementary note. (PDF)

S1 Fig Correlation between sample-specific gene function assignments at p-value 0.01 and p-value 0.001. For each function, we obtained the number of genes assigned that function in each sample, based on two different p-value thresholds 0.01 and 0.001. The figure shows the distribution of spearman correlation coefficients between the numbers of genes assigned a function based on the two p-values. The mean spearman correlation across all functions was 0.949.
(TIF)

S2 Fig Clustering quality. The figure shows the quality of the clustering (estimated by normalized Dunn index) for varying number (*k*) clusters. We chose *k* = 10 for our analyses, as the quality is relatively stabilized at that value.
(TIF)

S1 Table List of top 50 cancer-associated gained and top 50 cancer-associated lost functions. 3^rd^ and 4^th^ column: Normalized *Δ*_*f*_ is the net difference (gain or loss) divided by the number of genes annotated by *f* in the static PIN. Log fold change measures the log of the ratio of average number of expressed genes annotated by *f* in cancer and normal samples.
(XLSX)

S2 Table Table of sample-specific network statistics. The following table lists for each cancer sample (denoted by TCGA sample barcode), the number of genes considered to be expressed, the number of connected components and of the 1184 functions considered, the number that span multiple connected components.
(XLSX)

S3 Table Table of results depicting association between cancer-associated functional gain and loss with patient survival across different thresholds. Table lists the Fisher tests (columns 1–4) assessing the association of patient survival risk with predicted net gain or loss of functional activity in cancer (Δ_*f*_) for *θ* = 2 to 10 and additionally for *θ* = 2 and FDR < 0.1. The last column lists the spearman correlation values between Δ_*f*_ and function-wise the cox regression coefficient β for survival risk. Refer to main text for further details.
(XLSX)

S4 Table Table of results depicting survival predictive power of diffusion based approach versus annotation based approach. Table lists the 10-fold cross-validated C-indices (Column 1–2) of the two multivariate Cox regression models using the diffusion-based and annotation-based functional activity profiles of 24 case and 24 control functions respectively used as features for each model and repeated for *θ* = 2 to 10 and additionally for *θ* = 2 and FDR < 0.1. Columns 4,5 list the 10-fold cross validated C-indices of the diffusion and annotation based approach with pathway information incorporated. Columns 3 and 5 list the p-values associated with the differences in the C-indices of the two models.
(XLSX)

S5 Table Table of subtype and survival prediction results on METABRIC dataset in the absence of Netpath pathways as features. The table below displays the survival and subtype prediction accuracies of diffusion and annotation based functional profiles using as features the top cancer associated functions derived from TCGA and NetPath pathways as described in the Results section.
(XLSX)

S6 Table Table of top cancer associated functions. This table lists the top 24 (top and bottom 1%) functions ranked by *Δ*_*f*_ and likewise top 24 ranked by standard Gene set enrichment analysis based on differential expression.
(XLSX)

## References

[1] Barabási A-L, Oltvai ZN. Network biology: understanding the cell’s functional organization. Nat Rev Genet. 2004;5: 101–113. doi: 10.1038/nrg1272 14735121 10.1038/nrg1272

[2] Alon U. Biological networks: the tinkerer as an engineer. Science. 2003;301: 1866–7. doi: 10.1126/science.1089072 14512615 10.1126/science.1089072

[3] Lee I, Blom UM, Wang PI, Shim JE, Marcotte EM. Prioritizing candidate disease genes by network-based boosting of genome-wide association data. Genome Res. 2011;21: 1109–1121. doi: 10.1101/gr.118992.110 21536720 10.1101/gr.118992.110 PMC3129253

[4] Marcotte EM, Pellegrini M, Thompson MJ, Yeates TO, Eisenberg D. A combined algorithm for genome-wide prediction of protein function. Nature. Macmillian Magazines Ltd.; 1999;402: 83–86. Available: doi: 10.1038/47048 10573421 10.1038/47048

[5] Sharan R, Ulitsky I, Shamir R. Network-based prediction of protein function. Mol Syst Biol. 2007;3: 88 doi: 10.1038/msb4100129 17353930 10.1038/msb4100129 PMC1847944

[6] Sharan R, Ideker T. Modeling cellular machinery through biological network comparison. Nat Biotechnol. 2006;24: 427–433. doi: 10.1038/nbt1196 16601728 10.1038/nbt1196

[7] Stuart JM, Segal E, Koller D, Kim SK. A Gene-Coexpression Network for Global Discovery of Conserved Genetic Modules. Science (80-). 2003;302: 249–255. doi: 10.1126/science.1087447 12934013 10.1126/science.1087447

[8] Carroll AP, Tooney PA, Cairns MJ. Context-specific microRNA function in developmental complexity. Journal of Molecular Cell Biology. 2013 pp. 73–84. doi: 10.1093/jmcb/mjt004 23362311 10.1093/jmcb/mjt004

[9] Fossat N, Ip CK, Jones VJ, Studdert JB, Khoo P-L, Lewis SL, et al Context-specific function of the LIM homeobox 1 transcription factor in head formation of the mouse embryo. Development. 2015;142: 2069 LP-2079. Available: http://dev.biologists.org/content/142/11/2069.abstract 10.1242/dev.120907 25977363

[10] Kuntz SG, Williams BA, Sternberg PW, Wold BJ. Transcription factor redundancy and tissue-specific regulation: Evidence from functional and physical network connectivity. Genome Res. 2012;22: 1907–1919. doi: 10.1101/gr.133306.111 22730465 10.1101/gr.133306.111 PMC3460186

[11] Löhr U, Pick L. Cofactor-interaction motifs and the cooption of a homeotic Hox protein into the segmentation pathway of Drosophila melanogaster. Curr Biol. 2005;15: 643–649. doi: 10.1016/j.cub.2005.02.048 15823536 10.1016/j.cub.2005.02.048

[12] Curtis C, Shah SP, Chin S, Turashvili G, Rueda OM, Dunning MJ, et al The genomic and transcriptomic architecture of 2, 000 breast tumours. Nature. 2012; 1–7. doi: 10.1038/nature10983 22522925 10.1038/nature10983 PMC3440846

[13] van de Vijver MJ, He YD, van’t Veer LJ, Dai H, Hart A a M, Voskuil DW, et al A Gene-Expression Signature As a Predictor of Survival in Breast Cancer. N Engl J Med. 2002;347: 1999–2009. doi: 10.1056/NEJMoa021967 12490681 10.1056/NEJMoa021967

[14] Chuang HY, Lee E, Liu YT, Lee D, Ideker T. Network-based classification of breast cancer metastasis. Mol Syst Biol. 2007;3: 140 Artn 140\rDoi doi: 10.1038/msb4100180 17940530 10.1038/msb4100180 PMC2063581

[15] Ergün A, Lawrence C a, Kohanski MA, Brennan T a, Collins JJ. A network biology approach to prostate cancer. Mol Syst Biol. 2007;3: 82 doi: 10.1038/msb4100125 17299418 10.1038/msb4100125 PMC1828752

[16] Pujana MA, Han J-DJ, Starita LM, Stevens KN, Tewari M, Ahn JS, et al Network modeling links breast cancer susceptibility and centrosome dysfunction. Nat Genet. Nature Publishing Group; 2007;39: 1338–1349. Available: doi: 10.1038/ng.2007.2 17922014 10.1038/ng.2007.2

[17] Collins FS. The Cancer Genome Atlas (TCGA). Online. 2007. pp. 1–17.

[18] Schaefer MH, Fontaine JF, Vinayagam A, Porras P, Wanker EE, Andrade-Navarro MA. Hippie: Integrating protein interaction networks with experiment based quality scores. PLoS One. 2012;7 doi: 10.1371/journal.pone.0031826 22348130 10.1371/journal.pone.0031826 PMC3279424

[19] Vanunu O, Magger O, Ruppin E, Shlomi T, Sharan R. Associating genes and protein complexes with disease via network propagation. PLoS Comput Biol. 2010;6 doi: 10.1371/journal.pcbi.1000641 20090828 10.1371/journal.pcbi.1000641 PMC2797085

[20] Barshir R, Basha O, Eluk A, Smoly IY, Lan A, Yeger-Lotem E. The TissueNet database of human tissue protein-protein interactions. Nucleic Acids Res. 2013;41: D841–4. doi: 10.1093/nar/gks1198 23193266 10.1093/nar/gks1198 PMC3531115

[21] Magger O, Waldman YY, Ruppin E, Sharan R. Enhancing the Prioritization of Disease-Causing Genes through Tissue Specific Protein Interaction Networks. PLoS Comput Biol. 2012;8 doi: 10.1371/journal.pcbi.1002690 23028288 10.1371/journal.pcbi.1002690 PMC3459874

[22] Maiato H, Logarinho E. Mitotic spindle multipolarity without centrosome amplification. Nat Cell Biol. 2014;16: 386–94. doi: 10.1038/ncb2958 24914434 10.1038/ncb2958

[23] Chi JT, Rodriguez EH, Wang Z, Nuyten DSA, Mukherjee S, Van De Rijn M, et al Gene expression programs of human smooth muscle cells: Tissue-specific differentiation and prognostic significance in breast cancers. PLoS Genet. 2007;3: 1770–1784. doi: 10.1371/journal.pgen.0030164 17907811 10.1371/journal.pgen.0030164 PMC1994710

[24] Vogelstein B, Papadopoulos N, Velculescu VE, Zhou S, Diaz LA Jr., Kinzler KW. Cancer Genome Landscapes. Science (80-). 2013;339: 1546–1558. doi: 10.1126/science.1235122 23539594 10.1126/science.1235122 PMC3749880

[25] Bamford S, Dawson E, Forbes S, Clements J, Pettett R, Dogan A, et al The COSMIC (Catalogue of Somatic Mutations in Cancer) database and website. Br J Cancer. 2004;91: 355–8. doi: 10.1038/sj.bjc.6601894 15188009 10.1038/sj.bjc.6601894 PMC2409828

[26] Zhao L, Vogt PK. Class I PI3K in oncogenic cellular transformation. Oncogene. 2008;27: 5486–5496. doi: 10.1038/onc.2008.244 18794883 10.1038/onc.2008.244 PMC2757120

[27] Fernández-Medarde A, Santos E. Ras in cancer and developmental diseases. Genes Cancer. 2011;2: 344–58. doi: 10.1177/1947601911411084 21779504 10.1177/1947601911411084 PMC3128640

[28] Oren M, Rotter V. Mutant p53 gain-of-function in cancer. Cold Spring Harbor perspectives in biology. 2010 doi: 10.1101/cshperspect.a001107 20182618 10.1101/cshperspect.a001107 PMC2828285

[29] Pencina, Michael J and D’Agostino RB. Overall C as a measure of discrimination in survival analysis: model specific population value and confidence interval estimation. Stat Med. 2004;23: 2109–2123. doi: 10.1002/sim.1802 15211606 10.1002/sim.1802

[30] Kandasamy K, Mohan SS, Raju R, Keerthikumar S, Kumar GSS, Venugopal AK, et al NetPath: a public resource of curated signal transduction pathways. Genome Biol. 2010;11: R3 doi: 10.1186/gb-2010-11-1-r3 20067622 10.1186/gb-2010-11-1-r3 PMC2847715

[31] Gaujoux R, Seoighe C. A flexible R package for nonnegative matrix factorization. BMC Bioinformatics. 2010;11: 367 doi: 10.1186/1471-2105-11-367 20598126 10.1186/1471-2105-11-367 PMC2912887

[32] Moerkens M, Zhang Y, Wester L, van de Water B, Meerman JH. Epidermal growth factor receptor signalling in human breast cancer cells operates parallel to estrogen receptor alpha signalling and results in tamoxifen insensitive proliferation. BMC Cancer. 2014;14: 283 doi: 10.1186/1471-2407-14-283 24758408 10.1186/1471-2407-14-283 PMC4021213

[33] Gee JM, Robertson JF, Gutteridge E, Ellis IO, Pinder SE, Rubini M, et al Epidermal growth factor receptor/HER2/insulin-like growth factor receptor signalling and oestrogen receptor activity in clinical breast cancer. Endocr Relat Cancer. 2005;12 Suppl 1: S99–S111. doi: 10.1677/erc.1.01005 16113104 10.1677/erc.1.01005

[34] Nicholson RI, Johnston SR. Endocrine therapy—current benefits and limitations. Breast Cancer Res Treat. 2005;93 Suppl 1: S3–10. doi: 10.1007/s10549-005-9036-4 16247594 10.1007/s10549-005-9036-4

[35] Raju R, Balakrishnan L, Nanjappa V, Bhattacharjee M, Getnet D, Muthusamy B, et al A comprehensive manually curated reaction map of RANKL/RANK-signaling pathway. Database J Biol Databases Curation. Oxford University Press; 2011;2011: bar021 doi: 10.1093/database/bar021 21742767 10.1093/database/bar021 PMC3170171

[36] Jones DH, Nakashima T, Sanchez OH, Kozieradzki I, Komarova S V, Sarosi I, et al Regulation of cancer cell migration and bone metastasis by RANKL. Nature. 2006;440: 692–6. doi: 10.1038/nature04524 16572175 10.1038/nature04524

[37] James J, Evans A, Pinder S, Gutteridge E, Cheung K, Chan S, et al Bone metastases from breast carcinoma: histopathological–radiological correlations and prognostic features. Br J Cancer. 2003;89: 660–665. doi: 10.1038/sj.bjc.6601198 12915874 10.1038/sj.bjc.6601198 PMC2376918

[38] Canon J, Bryant R, Roudier M, Branstetter DG, Dougall WC. RANKL inhibition combined with tamoxifen treatment increases anti-tumor efficacy and prevents tumor-induced bone destruction in an estrogen receptor-positive breast cancer bone metastasis model. Breast Cancer Res Treat. Springer; 2012;135: 771–780. doi: 10.1007/s10549-012-2222-2 22926264 10.1007/s10549-012-2222-2

[39] Creixell P, Reimand J, Haider S, Wu G, Shibata T, Vazquez M, et al Pathway and network analysis of cancer genomes. Nat Methods. 2015;2: 1–6. doi: 10.1038/NMETH 10.1038/nmeth.3440 PMC4717906 26125594

[40] Bossi A, Lehner B. Tissue specificity and the human protein interaction network. Mol Syst Biol. 2009;5: 260 doi: 10.1038/msb.2009.17 19357639 10.1038/msb.2009.17 PMC2683721

[41] Komurov K, White M. Revealing static and dynamic modular architecture of the eukaryotic protein interaction network. Mol Syst Biol. 2007;3: 110 doi: 10.1038/msb4100149 17453049 10.1038/msb4100149 PMC1865589

[42] Taylor IW, Linding R, Warde-Farley D, Liu Y, Pesquita C, Faria D, et al Dynamic modularity in protein interaction networks predicts breast cancer outcome. Nat Biotechnol. 2009;27: 199–204. doi: 10.1038/nbt.1522 19182785 10.1038/nbt.1522

[43] Lui TW, Tsui NB, Chan LW, Wong CS, Siu PM, Yung BY. DECODE: an integrated differential co-expression and differential expression analysis of gene expression data. BMC Bioinformatics. 2015;16: 182 doi: 10.1186/s12859-015-0582-4 26026612 10.1186/s12859-015-0582-4 PMC4449974

[44] Zhu L, Ding Y, Chen CY, Wang L, Huo Z, Kim S, et al MetaDCN: Meta-analysis framework for differential co-expression network detection with an application in breast cancer. Bioinformatics. 2017;33: 1121–1129. doi: 10.1093/bioinformatics/btw788 28031185 10.1093/bioinformatics/btw788 PMC6041767

[45] Gillis J, Pavlidis P. “Guilt by association” is the exception rather than the rule in gene networks. PLoS Comput Biol. 2012;8 doi: 10.1371/journal.pcbi.1002444 22479173 10.1371/journal.pcbi.1002444 PMC3315453

[46] Luscombe NM, Babu MM, Yu H, Snyder M, Teichmann S a, Gerstein M. Genomic analysis of regulatory network dynamics reveals large topological changes. Nature. 2004;431: 308–312. doi: 10.1038/nature02782 15372033 10.1038/nature02782

[47] Barshir R, Basha O, Eluk A, Smoly IY, Lan A, Yeger-Lotem E. The TissueNet database of human tissue protein-protein interactions. Nucleic Acids Res. 2013;41 doi: 10.1093/nar/gks1198 23193266 10.1093/nar/gks1198 PMC3531115

[48] Erten S, Bebek G, Ewing RM, Koyutürk M. DADA: Degree-Aware Algorithms for Network-Based Disease Gene Prioritization. BioData Min. 2011;4: 19 doi: 10.1186/1756-0381-4-19 21699738 10.1186/1756-0381-4-19 PMC3143097

[49] Mermel CH, Schumacher SE, Hill B, Meyerson ML, Beroukhim R, Getz G. GISTIC2.0 facilitates sensitive and confident localization of the targets of focal somatic copy-number alteration in human cancers. Genome Biol. 2011;12: R41 doi: 10.1186/gb-2011-12-4-r41 21527027 10.1186/gb-2011-12-4-r41 PMC3218867

[50] Hackett NR, Butler MW, Shaykhiev R, Salit J, Omberg L, Rodriguez-Flores JL, et al RNA-Seq quantification of the human small airway epithelium transcriptome. BMC Genomics. 2012;13: 82 doi: 10.1186/1471-2164-13-82 22375630 10.1186/1471-2164-13-82 PMC3337229

[51] Zhou D, Bousquet O, Lal TN, Weston J, Schölkopf B. Learning with local and global consistency. Adv neural …. 2004;1: 595–602. citeulike-article-id:922481

[52] Harris MA, Clark J, Ireland A, Lomax J, Ashburner M, Foulger R, et al The Gene Ontology (GO) database and informatics resource. Nucleic Acids Res. 2004;32: D258–61. doi: 10.1093/nar/gkh036 14681407 10.1093/nar/gkh036 PMC308770

[53] Haibe-Kains B, Desmedt C, Sotiriou C, Bontempi G. A comparative study of survival models for breast cancer prognostication based on microarray data: Does a single gene beat them all? Bioinformatics. 2008;24: 2200–2208. doi: 10.1093/bioinformatics/btn374 18635567 10.1093/bioinformatics/btn374 PMC2553442

[54] Handl J, Knowles J, Kell DB. Computational cluster validation in post-genomic data analysis. Bioinformatics. 2005 pp. 3201–3212. doi: 10.1093/bioinformatics/bti517 15914541 10.1093/bioinformatics/bti517

