# Supplementary material for: A network diffusion approach to inferring sample-specific function reveals functional changes associated with breast cancer
Source: PLoS Comput Biol. 2017 Nov 30;13(11):e1005793. doi: 10.1371/journal.pcbi.1005793 (PMC5708603; doi:10.1371/journal.pcbi.1005793)
Supplement: S1 Text — (PDF) [file pcbi.1005793.s001.pdf]

Supplementary Note

Approximating null distributions

To determine the empirical p-values of the diffused raw score for each gene in each sample, we would require to store and read a large number of sample-specific null distribution tables. To save on time and space, we approximate the empirical sample-specific null distribution for each gene using a normal distribution that is parameterized by the mean and standard deviation of the empirical distribution. In this way, we only need to store a list of empirical mean and standard deviation pairs for each gene and sample (each pair corresponds to a null distribution). Since this approximation technique is independent of the input network, we demonstrate its robustness on the static human network as follows. We first partition all 1184 functions into 19 bins by seed size (bin 1 (seed size range): [50,69], … bin 19: [490-509]). This results in 19 null distribution tables, one for each bin. Based on which bin the diffused function lies in, we then estimate two p-values for each gene by reading the corresponding null distribution table for that bin. The first p-value is estimated directly from the table. Let this be called the empirical p-value. The second is obtained from the approximated normal distribution. Let this be called the analytical p-value. It is easy to see that the analytical p-values can be computed to a higher precision than the empirical p-values. Then, for each function, from the genes that get assigned based on the analytical p-value threshold of 0.01, we measure the fraction of genes that were assigned that function using the empirical p-value threshold of 0.01. Since on average 86% of all genes assigned a function analytically (from analytical p-value) also are assigned that function empirically (from empirical p-value) in the static network, the normal approximation is robust for the purposes of further downstream analyses.

Estimating the overall FDR for gain or loss of function and comment on robustness

We find that we make in total 42356 discoveries on the real data as opposed to 17848 discoveries when we randomly shuffle sample labels when estimating gain or loss of function at $\theta = 10$, and p-value < 0.05. Assuming all false discoveries were among the discoveries we made, empirical FDR≤42%. On the other hand, when we alternatively apply multiple testing correction on each function for the number of genes tested, which

corresponds to the setting of $\theta = 2$ and FDR < 0.1, we observe that we make 60706 discoveries on the real data as opposed to 43 after random shuffling of sample labels. This yields an empirical FDR ~ 0.07% in support of our approach. Nonetheless, our conclusions following all downstream analyses on the TCGA data are similar in these two settings.

Comparison with gene set enrichment analysis

We perform the gene set enrichment analysis as follows. We find differentially expressed genes between the two cohorts based on a 2 sided Wilcoxon rank sum test. A gene was considered differentially expressed if its adjusted p-value is < 0.05 (based on Benjamini Hochberg method) and absolute log fold change in median expression > 1.5. We thus identify 2969 differentially expressed genes between the healthy and cancer cohort. We then perform a gene set enrichment analysis using as gene sets the 1184 functions we used, using a Hypergeometric test. The functions are ranked in ascending order based on their adjusted p-values to reveal the top 24 functions based on GSEA. These are likewise compared with the top 24 (top and bottom 1%) based on diffusion. As seen from S6 Table, the 24 functions based on diffusion are not revealed by GSEA and vice-versa.

Moreover, we compute sample specific Z-scores based on comparison of sample-specific gene expression with the normal expression distribution of that gene across samples and then compute the mean Z-score for each functional gene-set in each sample. Using this as an alternative for functional activity profiles, we re-run the survival prediction analysis using the top 24 GSEA functions in conjunction with NetPath features. We get a C-index of 0.63, which is not significantly different from 0.62, obtained using our approach. However, the two feature sets of cancer-associated functions are starkly different. While one is based on expression variability of genes, the other is based on PPI variability, hence validating our original hypothesis that genes may also exhibit functional changes in addition to expression based changes that are linked with cancer.
