# Supplementary figures and images for: A network diffusion approach to inferring sample-specific function reveals functional changes associated with breast cancer
Source: PLoS Comput Biol. 2017 Nov 30;13(11):e1005793. doi: 10.1371/journal.pcbi.1005793 (PMC5708603; doi:10.1371/journal.pcbi.1005793)

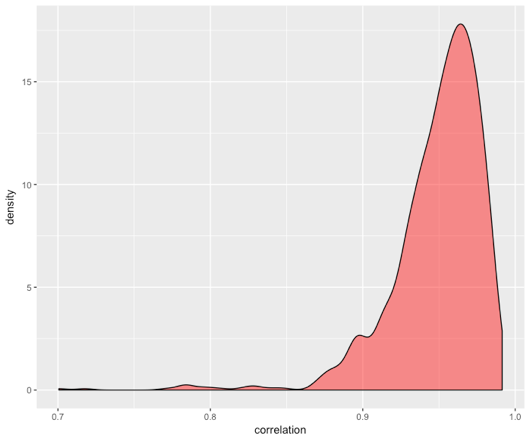

Supplement: S1 Fig — For each function, we obtained the number of genes assigned that function in each sample, based on two different p-value thresholds 0.01 and 0.001. The figure shows the distribution of spearman correlation coefficients between the numbers of genes assigned a function based on the two p-values. The mean spearman correlation across all functions was 0.949. (TIF) [file pcbi.1005793.s002.tif]

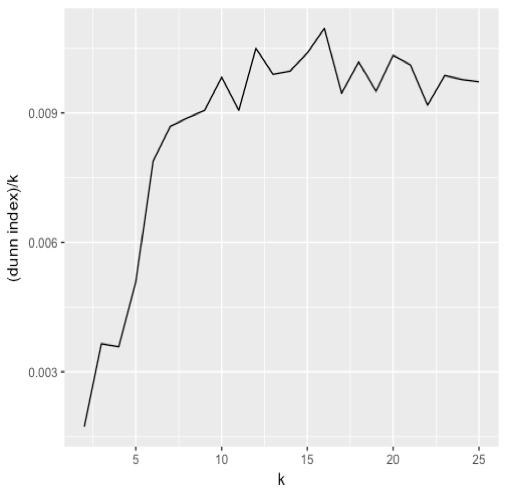

Supplement: S2 Fig — The figure shows the quality of the clustering (estimated by normalized Dunn index) for varying number (k) clusters. We chose k = 10 for our analyses, as the quality is relatively stabilized at that value. (TIF) [file pcbi.1005793.s003.tif]
